# Mean platelet volume is associated with serum 25-hydroxyvitamin D concentrations in patients with stable coronary artery disease

**DOI:** 10.1007/s00380-018-1182-9

**Published:** 2018-05-03

**Authors:** Ilona Korzonek-Szlacheta, Bartosz Hudzik, Justyna Nowak, Janusz Szkodzinski, Jolanta Nowak, Mariusz Gąsior, Barbara Zubelewicz-Szkodzinska

**Affiliations:** 1 Department of Nutrition-Related Disease Prevention, School of Public Health, Medical University of Silesia, Bytom, Poland; 2 Third Department of Cardiology, Silesian Center for Heart Disease, School of Medicine with the Division of Dentistry in Zabrze, Medical University of Silesia, Curie-Sklodowska 9, 41-800 Zabrze, Poland

**Keywords:** Mean platelet volume, Vitamin D, 25(OH)D, Coronary artery disease

## Abstract

There is little published data on the association of platelet function and 25(OH)D concentration. We investigated the associations between mean platelet volume (MPV) and 25(OH)D concentration in patients with stable coronary artery disease. Study population was divided into three groups: group 1—25(OH)D < 10 ng/mL (*N* = 22), group 2—25(OH)D 10–20 ng/mL (*N* = 42), and group 3—25(OH)D > 20 ng/mL (*N* = 14). Study groups shared similar demographics. MPV values were the highest in group 1, moderate in group 2, and the lowest in group 3 (11.1 vs 10.4 vs 9.8 fL *P* < 0.001). There was a negative correlation between MPV and 25(OH)D (*R* = − 0.38, *P* = 0.001). ROC analysis demonstrated a moderate predictive value (AUC 0.70) in identifying the discrimination thresholds of MPV (> 10.5 fL) for vitamin D deficiency and a weak predictive value (AUC 0.65) in identifying the discrimination thresholds of 25(OH)D concentration (≤ 15.5 ng/mL) for the presence of large platelets (MPV over the upper limit of normal). In conclusion, even though the effect of vitamin D on platelet size and function is probably multifactorial, our study provides further evidence linking vitamin D to thrombosis and hemostasis. Platelets are another potential element through which vitamin D deficiency could exert adverse cardiovascular outcomes.

## Introduction

Platelets are key elements in various mechanisms in the human body including hemostasis and thrombosis, immunity, and inflammation [1–4]. More importantly, platelet activation is one of the hallmark features of atherothrombosis [5]. Following activation, platelet responses are relatively uniform with a change in shape, size, aggregation, cell expression, and release of a range of cytokines [6]. Platelet size has been reported to reflect its activity. Larger-size platelets are metabolically and enzymatically more active [7]. However, assays of platelet function may be very time consuming, expensive, and technically difficult. On the contrary, mean platelet volume (MPV), which is readily available in clinical settings, has been linked to increased rate or risk of coronary artery disease (CAD), acute coronary syndromes (ACS), hypertension, stroke and to unfavorable outcomes including mortality [8, 9]. Elevated MPV has been associated with unfavorable outcomes following percutaneous coronary intervention (PCI) in CAD, higher rates of myocardial infarction (MI) and higher mortality rates following MI. Therefore, increased MPV is a potentially useful prognostic biomarker in patients with cardiovascular disease [9–12].

Vitamin D has been considered to be a significant component of bone and mineral metabolism [13]. In addition, vitamin D receptors are present on many other organs, including, but not limited to, the pancreas, large and small intestines, muscles, the myocardium, endothelial cells, nervous and immune system [14]. In fact, the best indicator of vitamin D status is the serum 25-hydroxyvitamin D [25(OH)D] concentration, because it reflects both dietary intake from vitamin D and cutaneous synthesis of vitamin D [15].

Vitamin D deficiency and insufficiency are highly prevalent in the general population worldwide [16]. The main reasons for such a phenomenon are greater sun protection, lack of outdoor sun exposure, and increase in population body mass index. Severe vitamin D deficiency causes rickets or osteomalacia. Less severe forms have been linked to other serious consequences: increased risk of type 1 and type 2 diabetes mellitus, multiple sclerosis, rheumatoid arthritis, hypertension, cardiovascular disease, and cancer (e.g., colon, breast) [13, 14].

Adverse effects of vitamin D deficiency on hemostasis and thrombosis have increasingly been shown in in vitro and animal studies [17]. However, data from limited human studies are conflicting and raise a much debated issue of such associations [18, 19]. Vitamin D has been proved to diminish the expression of plasminogen activator inhibitor I (PAI-I), tissue factor, and thrombomodulin [20, 21]. There is little published data on the association of platelet function and 25(OH)D concentration. Moreover, there has been little detailed investigation on the link between MPV and vitamin D. Given the paucity of data on the effects of vitamin D on hemostasis and thrombosis and the numerous investigations on the cardiovascular effects of vitamin D, we sought to obtain data which may help to address these research gaps. This study set out to investigate the associations between MPV and 25(OH)D concentration in patients with stable CAD.

## Patients and methods

The study conforms to the Declaration of Helsinki and was approved by the institutional review board at the Medical University of Silesia. Seventy-eight patients with stable CAD (Canadian Cardiovascular Society functional class II) were enrolled. Stable CAD was defined as a history of documented myocardial infarction, prior coronary revascularization, chest pain with documented myocardial ischemia on non-invasive tests or coronary stenosis of > 50% proven by angiography. The patients needed to be stable for the prior 6 months without any change in functional status. Exclusion criteria included history of acute coronary syndrome within 12 months prior to enrollment, previous percutaneous coronary intervention within 12 months prior to enrollment, previous coronary artery bypass graft surgery within 12 months prior to enrollment, vitamin D/multivitamin supplements and/or calcium supplementation within 12 months prior to enrollment, P2Y12 inhibitor therapy, cancer (being under treatment and/or diagnosed with malignancies), chronic kidney disease—stage 3 or higher (baseline estimated glomerular filtration rate < 60 mL/min/1.73 m^2^), uncontrolled thyroid dysfunction, liver dysfunction (including viral hepatitis, cholestatic jaundice with bilirubin concentration > 1.5 mg/dL and/or alkaline phosphatase at least twice the upper limit of normal), calcium metabolic disorders, hormone replacement therapy, coexisting autoimmune disorders, acute infectious disease, chronic inflammatory disease, cancer, uncontrolled diabetes mellitus, ischemic or hemorrhagic stroke during 12 months before admission, glucocorticoids and/or androgens therapy, admission platelet count < 100 or > 450 × 10^3^/μL (to avoid any underlying platelet pathology), and lack of patient consent to participate.

Given seasonal variations in serum 25(OH)D concentrations, all blood samples were collected in the spring season. Venous blood samples were collected between 8 and 9 AM, and separated by centrifugation at 4 °C, 1800*×g* for 15 min. Samples were then stored at − 80 °C until further analysis. Serum 25-hydroxyvitamin D [25(OH)D] measurements were carried out using a commercial kit—Architect (Abbott Diagnostics, Lake Forest, IL, USA) 25OH Vitamin D chemiluminescence microparticle immunoassay (CMIA) in duplicates. Assay range was determined as 3.4–156.0 ng/mL. Measurements for each patient were made with the same kit to avoid inter-kit variability. The intra-assay coefficient of variation (%CV) was 2.7% and the inter-assay %CV was 3.5%. Precision error was < 8%.

Complete blood counts (CBC) were performed using the Sysmex XS1000i and XE2100 apparatus (Sysmex Corporation, Kobe, Japan). CBC platelet volume indices included: MPV and platelet distribution width (PDW). We used ethylenediaminetetraacetic acid (EDTA) for whole blood anticoagulation. To avoid any preanalytical influence of EDTA on MPV, all blood samples were tested within 1 h of collection. Studies indicate that MPV can be measured accurately by both methods of anticoagulation—EDTA and citrate—if analysis is performed within 1 h of sampling.

Based on serum 25(OH)D status, the study population was divided into three groups [22]: group 1—25(OH)D concentration of less than 10 ng/mL (*N* = 22), group 2—25(OH)D concentration of 10–20 ng/mL (*N* = 42), and group 3—25(OH)D concentration of more than 20 ng/mL (*N* = 14).

### Statistical analysis

Quantitative data are presented as medians and interquartile ranges (lower and upper quartiles). Qualitative data are presented as frequencies. The Shapiro–Wilk test was used to determine whether a random sample was normally distributed. Kruskal–Wallis ANOVA was used to test the differences between the three groups. The chi-square test with Yates’ correction and Fisher’s exact test was used to compare categorical variables. The relationship between vitamin D and platelet indices was evaluated by Spearman’s rank correlation coefficient. A receiver operating characteristic (ROC) analysis was performed to assess the ability of MPV to predict 25(OH)D deficiency [defined as 25(OH)D concentration less than 20 ng/mL] and to assess the ability of 25(OH)D concentration to predict increased platelet volume (defined as MPV greater than 10.9 fL). Furthermore, ROC analysis identified the optimal cutoff values of MPV and vitamin D.

## Results

**Fig. 1:**
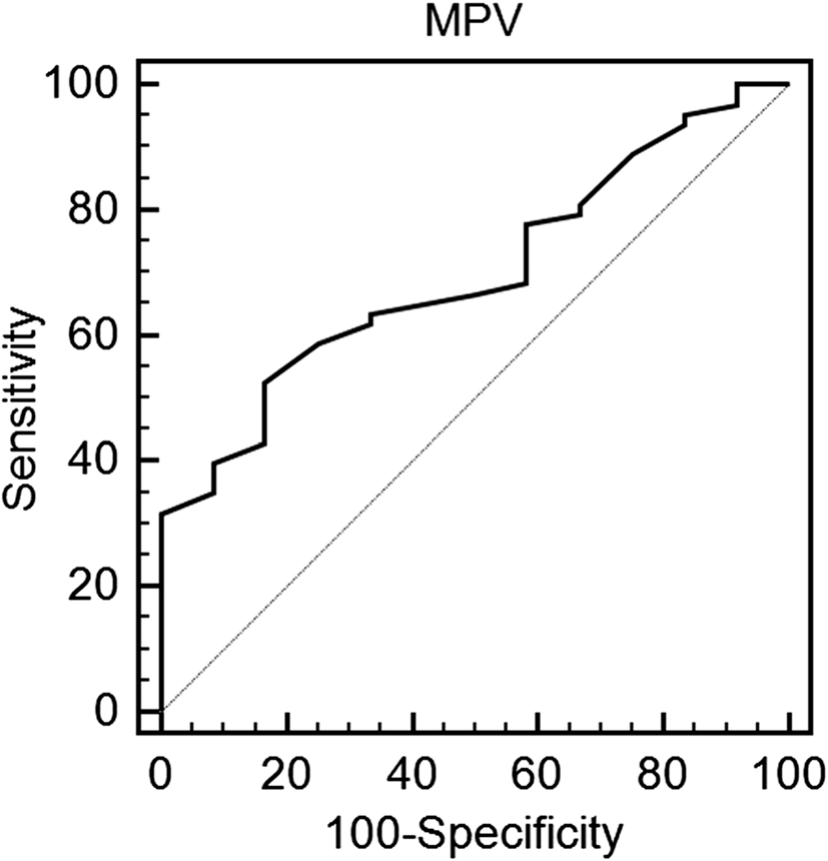
Receiver operating characteristics curve identifying the discrimination threshold of mean platelet volume for vitamin D deficiency

**Fig. 2:**
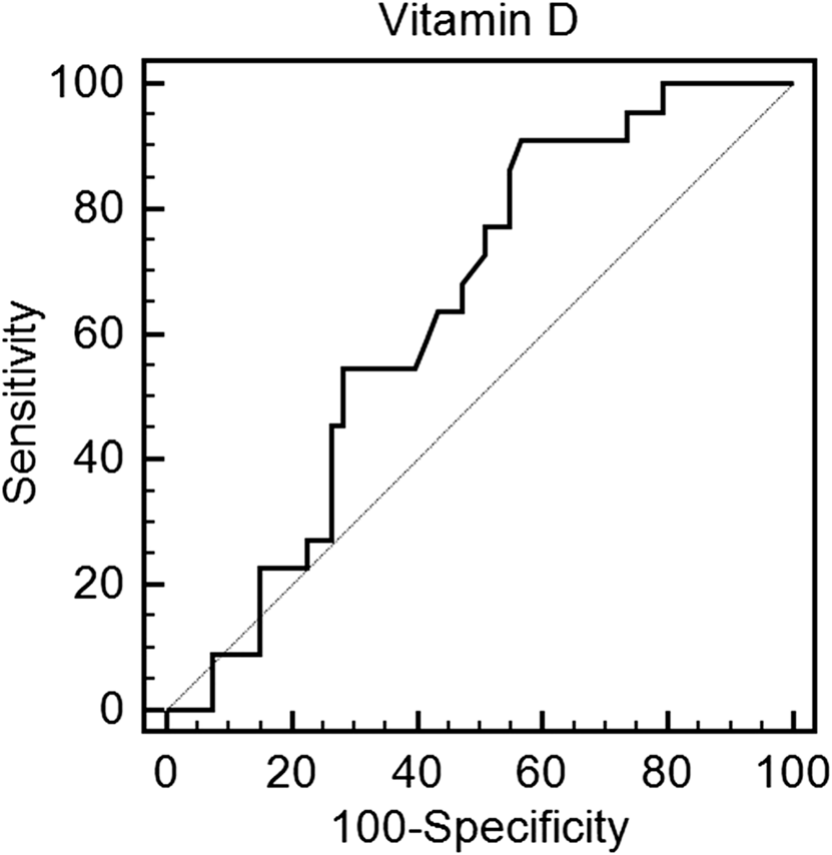
Receiver operating characteristics curve identifying the discrimination thresholds of 25(OH)D concentration for the presence of large platelets (mean platelet volume over the upper limit of normal)

All but one patient in the study population had vitamin D insufficiency or deficiency [25(OH)D concentration below 30 ng/mL]. 22 patients (28.2%) presented with severe vitamin D deficiency [25(OH)D concentration below 10 ng/mL], 42 patients (58.3%) presented with moderate vitamin D deficiency [25(OH)D concentration between 10 and 20 ng/mL], and 11 patients presented with vitamin D insufficiency [25(OH)D concentration between 20 and 30 ng/mL]. Table 1 displays main clinical features. Study groups shared similar demographics. Patients across all three study groups received similar medical therapy. Laboratory results are shown in Table 2. MPV values were the highest in group 1, moderate in group 2, and the lowest in group 3 (11.1 vs 10.4 vs 9.8 fL *P* < 0.001), whereas PDW values were the highest in group 1 and 2. Moreover, MPV to platelet count (MPV/PC) ratio was the highest among the two groups with vitamin D deficiency (group 1 and 2) in comparison with group 3. In addition, there was a trend towards a higher MPV to lymphocyte ratio (MPVLR) in patients with the lowest 25(OH)D concentrations. Notwithstanding, platelet to lymphocyte ratio (PLR) was similar in all three study groups. There was a negative correlation between MPV and 25(OH)D concentration (*R* = − 0.38, *P* = 0.001) (Table 3). Linear regression revealed that low 25(OH)D was independently associated with high MPV (*β* = − 0.18, *P* < 0.0001). ROC analysis demonstrated a moderate predictive value (AUC 0.70) in identifying the discrimination thresholds of MPV (> 10.5 fL) for vitamin D deficiency and a weak predictive value (AUC 0.65) in identifying the discrimination thresholds of 25(OH)D concentration (≤ 15.5 ng/mL) for the presence of large platelets (MPV over the upper limit of normal) (Table 4, Figs. 1, 2).

**Table 1:** Baseline clinical characteristics

	Group 1 (*n* = 22)	Group 2 (*n* = 42)	Group 3 (*n* = 14)	*P*
Age, years	79 (69–84)	78 (68–85)	77 (70–82)	0.6
Sex, men *n* (%)	4 (18.2)	17 (40.0)	5 (36.0)	0.07
Systemic hypertension *n* (%)	21 (95.0)	37 (90.6)	13 (92.8)	0.6
Hyperlipidemia *n* (%)	4 (18.2)	10 (23.8)	4 (28.6)	0.8
Diabetes mellitus *n* (%)	13 (60.0)	12 (28.6)	9 (63.3)	0.09
Prior myocardial infarction *n* (%)	1 (4.6)	9 (22.0)	4 (28.6)	0.1
BMI	25 (23–29)	29 (25–32)	27 (24–30)	0.3
Aspirin *n* (%)	22 (100)	42 (100)	14 (100)	1.0
Beta-blockers *n* (%)	14 (66.7)	35 (83.3)	10 (71.4)	0.3
ACE inhibitors *n* (%)	15 (71.4)	26 (61.9)	6 (42.8)	0.2
Statins *n* (%)	8 (38.1)	24 (57.1)	7 (50.0)	0.07

Values are presented as medians (interquartile range) or *n* (%)

**Table 2:** Laboratory findings

	Group 1 (*n* = 22)	Group 2 (*n* = 42)	Group 3 (*N* = 14)	*P*
Leucocytes (10^3^/mm^3^)	6.4 (4.8–8.6)	6.8 (5.5–8.2)	6.1 (4.7–7.1)	0.3
Erythrocytes (10^6^/mm^3^)	4.1 (3.8–4.6)	4.3 (3.9–4.6)	4.1 (3.9–4.3)	0.6
Lymphocytes (10^3^/mm^3^)	2.2 (1.3–3.4)	2.5 (1.6–3.6)	2.2 (1.7–2.6)	0.6
Hemoglobin (g/dL)	12.6 (12.1–13.8)	12.6 (11.7–13.4)	12.3 (11.3–13.1)	0.3
Hematocrit (%)	38 (36–42)	39 (36–41)	38 (37–39)	0.7
Platelets (10^3^/mm^3^)	218 (173–262)	208 (168–248)	230 (204–277)	0.3
Total cholesterol (mmol/L)	4.3 (3.8–5.5)	4.4 (3.6–5.2)	4.3 (3.7–5.1)	0.7
HDL cholesterol (mmol/L)	1.6 (1.1–2.0)	1.4 (1.1–1.7)	1.2 (1.1–1.3)	0.1
LDL cholesterol (mmol/L)	2.3 (1.3–3.6)	2.7 (1.7–3.0)	2.5 (1.8–3.2)	0.8
Triglycerides (mmol/L)	1.0 (0.9–1.6)	1.2 (0.9–14)	1.1 (0.9–1.9)	0.8
Serum creatinine (μmol/L)	68 (60–90)	78 (66–98)	75 (66–88)	0.16
Aspartate aminotransferase (AST) (U/L)	18 (15–20)	18 (15–21)	18 (14–19)	0.5
Alanine aminotransferase (ALT) (U/L)	15 (10–22)	15 (11–20)	16 (11–17)	0.7
MCV (fL)	92.5 (88.0–95.7)	91.5 (88.6–93.5)	91.4 (88.2–94.4)	0.3
MPV (fL)	11.1 (10.3–11.6)	10.4 (9.8–10.9)	9.8 (9.4–10.1)	< 0.001
PDW (fL)	14.2 (11.7–14.9)	14.0 (12.2–15.0)	11.9 (11.0–13.6)	0.007
Vitamin D (ng/mL)	8.1 (7.1–9.2)	13.8 (12.1–15.8)	23.4 (21.3–25.2)	< 0.001
MPV/PC	0.056 (0.041–0.063)	0.055 (0.040–0.061)	0.041 (0.030–0.051)	0.04
PLR	104.5 (75.4–140.5)	99.0 (67.6–121.9)	109.0 (91.5–130.3)	0.1
MPVLR	5.1 (3.6–6.7)	4.3 (3.3–5.5)	4.7 (4.1–5.1)	0.07

Values are presented as medians (interquartile range)

*HDL* high-density lipoprotein, *LDL* low-density lipoprotein, *MCV* mean corpuscular volume, *MPV* mean platelet volume, *MPVLR* mean platelet volume to lymphocyte ratio, *MPV/PC* mean platelet volume to platelet count ratio, *PDW* platelet distribution width, *PLR* platelet to lymphocyte ratio

**Table 3:** Correlations between 25-hydroxyvitamin D [25(OH)D] and platelet indices

	25(OH)D	
	Spearman *R*	*P*
Platelets	− 0.10	0.7
MPV	− 0.38	0.001
PDW	0.02	0.8
MPV/PC	− 0.06	0.6
PLR	− 0.01	0.9
MPVLR	− 0.05	0.4

*MPV* mean platelet volume, *MPVLR* mean platelet volume to lymphocyte ratio, *MPV/PC* mean platelet volume to platelet count ratio, *PDW* platelet distribution width, *PLR* platelet to lymphocyte ratio P-LCR platelet large cell ratio

**Table 4:** Receiver operating characteristics curves identifying the discrimination thresholds of mean platelet volume for vitamin D deficiency and 25(OH)D concentration for the presence of large platelets (mean platelet volume over the upper limit of normal)

	Cut off	AUC	95% CI	Sensitivity (%)	Specificity (%)	PPV (%)	NPV (%)	*P*
Vitamin D deficiency								
MPV	> 10.5	0.70	0.58–0.78	52	83	94	25	0.006
Large platelets (MPV over the upper limit of normal)								
25(OH)D	≤ 15.5	0.65	0.52–0.75	91	43	40	95	0.03

*AUC* area under the curve, *PPV* positive predictive value, *NPV* negative predictive value, *MPV* mean platelet volume

## Discussion

We have set out to investigate the link between MPV and 25(OH)D concentration. There are several key points of our study. First and foremost, vitamin D insufficiency or deficiency is common among patients with stable CAD. Second, MPV values increased with the decline in 25(OH)D concentration, while PDW values remained elevated in patients with vitamin D insufficiency and deficiency. In addition, we found that among all platelet volume indices, only MPV demonstrated a significant negative correlation with 25(OH)D concentration. And finally, MPV showed a moderate ability to predict vitamin D deficiency, while 25(OH)D concentration showed significant but weak ability to predict increased platelet volume.

Only few studies evaluated the associations between MPV and 25(OH)D. Cumhur Cure et al. examined 434 patients without chronic disease who were not taking vitamin D or calcium supplements and found that MPV in participants with 25(OH)D concentration of more than 20 ng/mL (7.5 ± 1.0 fL) was lower than that in participants with 25(OH)D concentration below 10 ng/mL (8.1 ± 1.1 fL, *P* < 0.001) and in participants with 25(OH)D concentration of between 10 and 20 ng/mL (7.9 ± 1.0 fL, *P* = 0.009) [22]. Similar results were obtained by Kebapcilar et al. who examined the relationship between mean platelet volume and low-grade systemic coagulation with vitamin D deficiency in primary ovarian insufficiency [23]. They reported that serum 25(OH)D was inversely correlated with MPV (*R* = − 0.528, *P* < 0.001), activated partial thromboplastin time (APTT) (*R* = − 0.344, *P* = 0.002), and D-dimer (*R* = − 0.425, *P* < 0.001). Thus, they indicated that vitamin D deficiency could be associated with hypercoagulability [23]. Our results are consistent with those of Cumhur Cure and Kebapcilar, and further support previous limited data into the area that link thrombosis and hemostasis with vitamin D.

As mentioned previously, platelet size has been reported to reflect its activity. Larger-size platelets are metabolically and enzymatically more active [7]. Interestingly, Verdoia et al. evaluated the impact of 25(OH)D concentrations on platelet function in patients treated with dual antiplatelet therapy (DAPT) [aspirin and P2Y12 receptor inhibitor (clopidogrel or ticagrelor)] [24]. The prevalence of high-residual platelet reactivity (HRPR) for aspirin was low (1.2%) and not conditioned by 25(OH)D levels [adjusted OR (95% CI) = 1.56 (0.71–3.5), *P* = 0.27]. HRPR with P2Y12 receptor inhibitors (clopidogrel or ticagrelor) was observed in 26% of patients, and the rate increased with lower 25(OH)D quartiles [37.3 vs 22.2 vs 24.4 vs 20.2%, *p* = 0.005, adjusted OR (95% CI) = 1.23 (1.02–1.49), *P* = 0.04] [24]. Thus, lower 25(OH)D levels could be associated with higher platelet reactivity and impaired effectiveness of P2Y12 receptor inhibitors (clopidogrel or ticagrelor), while not influencing the effectiveness of aspirin.

When considering platelets and vitamin D, we have to keep in mind that vitamin D receptor (VDR) has been recently found in platelets and thus megakaryocytopoiesis and platelet activation, which are calcium-dependent events, might be modulated by a mitochondrial non-genomic activity of VDR [25]. In vitro and in vivo experiments indicated that the vitamin D-VDR system plays a pivotal role in antithrombogenicity [26, 27]. Aihara et al. reported that activation of nuclear VDR elicits antithrombotic effects in vivo, and suggest that the VDR system could potentially play a physiological role in the maintenance of antithrombotic homeostasis [26, 27].

On the subject of advanced age and high rate of comorbid conditions in our study, it is noteworthy to emphasize that multimorbidity was demonstrated to influence MPV values as well as 25(OH)D concentrations. Hudzik et al. indicated that multimorbidity was associated with an increase in platelet volume indices. MPV values increased with the increasing number of comorbid conditions. Importantly, MPV values were elevated in a wide range of cardiovascular and non-cardiovascular diseases [28]. Meems et al. investigated the link between 25(OH)D concentration and the prevalence of multimorbidity in the general population [29]. They provided evidence that low levels of 25(OH)D were associated with higher prevalence of multimorbidity, especially in participants with 25(OH)D levels below 10 ng/mL.

And finally, inflammation may be an important biological mechanism through which low 25(OH)D concentrations are linked to elevated MPV. Studies suggest that higher levels of circulating cytokines in vitamin D-deficient patients could be held responsible for a higher platelet reactivity [30, 31]. Although, the effects of vitamin D deficiency on coagulation, fibrinolysis, and inflammation have been increasingly demonstrated in both in vitro and animal studies, the human studies remain inconclusive [17].

In conclusion, even though the effect of vitamin D on platelet size and function is probably multifactorial, our study provides further evidence linking vitamin D to thrombosis and hemostasis. Platelets are another potential element through which vitamin D deficiency could exert adverse cardiovascular outcomes.

### Study limitations

Our study needs to be viewed in light of its limitations. This study is a cross-sectional study: therefore, these analyses do not provide information about possible causality of vitamin D deficiency and increased thrombotic risk. Observational nature of the study cannot exclude a possible effect of any unmeasured factors on the observed associations. In addition, there is a seasonal change in 25(OH)D concentration; however, we avoided this problem by enrolling all patients in the spring season. Lower 25(OH)D concentrations during winter season arise from less sun exposure and lower amount of UV-B radiation reaching the skin resulting in lower cutaneous vitamin D3 synthesis [32, 33]. More importantly despite these limitations, we applied a strict set of exclusion criteria to obtain a homogenous population with respect to conditions and medical therapy that may have influenced MPV values and 25(OH)D concentrations. And finally, the relatively small number of patients enrolled might have rendered some differences insignificant. Further large-scale studies are warranted in the field of vitamin D and thrombosis/hemostasis.
